# Attrition when providing antiretroviral treatment at CD4 counts >500cells/μL at three government clinics included in the HPTN 071 (PopART) trial in South Africa

**DOI:** 10.1371/journal.pone.0195127

**Published:** 2018-04-19

**Authors:** Peter Bock, Geoffrey Fatti, Nathan Ford, Karen Jennings, James Kruger, Colette Gunst, Françoise Louis, Nelis Grobbelaar, Kwame Shanaube, Sian Floyd, Ashraf Grimwood, Richard Hayes, Helen Ayles, Sarah Fidler, Nulda Beyers, on behalf of the HPTN 071 (PopART) trial team

**Affiliations:** 1 Desmond Tutu TB Centre, Department of Paediatrics and Child Health, Faculty of Medicine and Health Sciences, Stellenbosch University, Tygerberg Campus, Western Cape, South Africa; 2 Kheth’ Impilo, Foreshore, Cape Town, South Africa; 3 Centre for Infectious Disease Epidemiology and Research, University of Cape Town, Cape Town, South Africa; 4 City of Cape Town Health Services, Foreshore, Cape Town, South Africa; 5 Western Cape Department of Health, HIV Treatment & PMTCT programme, Cape Town, South Africa; 6 Western Cape Department of Health Cape Winelands District Brewelskloof Hospital, Worcester, South Africa; 7 Stellenbosch University Division of Family Medicine and Primary Health Care, Faculty of Medicine and Health Sciences,Tygerberg Campus, Western Cape, South Africa; 8 ANOVA Healthcare, Paarl, South Africa; 9 Zambart, University of Zambia, Ridgeway Campus, Lusaka, Zambia; 10 Department of Infectious Disease Epidemiology, London School of Hygiene and Tropical Medicine, London, United Kingdom; 11 Department of Clinical Research, London School of Hygiene and Tropical Medicine, London, United Kingdom; 12 Department of Medicine, Imperial College London, St Mary’s Campus, London, United Kingdom; San Antonio Military Medical Center, UNITED STATES

## Abstract

**Introduction:**

WHO recommends antiretroviral treatment (ART) for all HIV-positive individuals. This study evaluated the association between baseline CD4 count and attrition in a cohort of HIV positive adults initiating ART at three department of health (DOH) clinics routinely providing ART at baseline CD4 counts >500cells/μL for the HPTN 071 (PopART) trial.

**Methods:**

All clients attending the DOH clinics were managed according to standard care guidelines with the exception that those starting ART outside of pertinent local guidelines signed research informed consent. DOH data on all HIV-positive adult clients recorded as having initiated ART between January 2014 and November 2015 at the three study clinics was analysed. Attrition, included clients lost to follow up or died, and was defined as ‘being three or more months late for an antiretroviral pharmacy pick-up appointment’. All clients were followed until attrition, transfer out or end May 2016.

**Results:**

A total of 2423 clients with a median baseline CD4 count of 328 cells/μL (IQR 195–468) were included of whom 631 (26.0%) experienced attrition and 140 (5.8%) were TFO. Attrition was highest during the first six months of ART (IR 38.3/100 PY; 95% CI 34.8–42.1). Higher attrition was found amongst those with baseline CD4 counts > 500 cells/μL compared to those with baseline CD4 counts of 0–500 cells/μL (aHR 1.26, 95%CI 1.05 to 1.52) This finding was confirmed on subset analyses when restricted to individuals non-pregnant at baseline and when restricted to individuals with follow up of > 12months.

**Conclusions:**

Attrition in this study was high, particularly during the first six months of treatment. Attrition was highest amongst clients starting ART at baseline CD4 counts > 500 cells/μL. Strategies to improve retention amongst ART clients, particularly those starting ART at baseline CD4 counts >500cells/μL, need strengthening. Improved monitoring of clients moving in and out of ART care and between clinics will assist in better understanding attrition and ART coverage in high burden countries.

## Introduction

There are 36.7 million HIV positive individuals and 19.5 million people on antiretroviral treatment (ART) worldwide [1]. UNAIDS has set global HIV treatment targets of 90:90:90; 90% of HIV positive individuals knowing their HIV status, of which 90% are on ART, of which 90% are virally suppressed [2]. To achieve the sustained viral suppression required to prevent progression to AIDS disease in HIV positive individuals and to limit onward viral transmission of HIV, high levels of retention in ART care and adherence to medication are required [3, 4].

Retention in ART programmes in high burden settings is extremely challenging. A recent systematic review, which included 1.5 million participants from African and Asian programmatic studies (75% from Africa), the majority of whom started ART at baseline CD4 counts < 200cells/μL, found 17% and 26% of individuals on ART lost to follow up or died (attrition) at 12 months and at 24 months respectively [5]. The association between baseline CD4 count and attrition varies, with some programmatic studies having reported decreased, [6] and some showing increased attrition at higher CD4 counts [7–9]. The median baseline CD4 counts in these published studies, however, was low with a high proportion of individuals starting ART at CD4 counts <200cells/μL and mortality may have contributed extensively to attrition amongst individuals with lower CD4 counts.

Following results of the START and TEMPRANO randomised control trials (RCTs) in 2015 [10, 11], WHO guidelines have recommended ART for all PLHIV regardless of CD4 count [12]. There are, however, very limited published data evaluating the impact of routine provision of ART at CD4 counts > 500cells/μL on attrition from ART programmes in high burden settings. Concerns also remain that increased numbers of clients starting ART at baseline CD4 counts > 500cell/μL, when clinically well, may be associated with increased attrition [13]. This study evaluated the association between baseline CD4 count > 500cells/μL and attrition in a cohort of adults initiating ART regardless at three department of health (DOH) clinics in the Western Cape Province, South Africa.

## Methods

### Study setting

This study was conducted at three DOH primary health care (PHC) clinics included in the ‘Population effect of antiretroviral therapy to reduce HIV incidence’ HPTN 071 (PopART) trial in the Western Cape (WC), South Africa. A full description of the HPTN 071 (PopART) trial design has been published [14]. The communities surrounding the three study clinics received the full HPTN 071 (PopART) intervention which consisted of household delivery of an HIV combination prevention package, including HIV rapid testing in the house by community HIV care providers (CHiPs), referral to the clinics and active linkage to ART care. CHiPs routinely visited clients annually with more intensive follow up when clinically indicated e.g. after HIV diagnosis [14].

Two clinics were located in the metro district (Metro 1 and 2) and one in a rural district (Rural 1). These study clinics offered ART regardless of CD4 count for all HIV positive clients aged 18 or older. During the study period for standard care at other clinics DOH ART guidelines recommended ART initiation at baseline CD4 count ≤ 350 cells/μL until January 2015 and thereafter at baseline CD4 count ≤500cells/μL [15]. All clients attending the study clinics received standard care as per DOH ART guidelines with the exception that ART was provided to all HIV positive individuals and individuals starting ART outside of pertinent DOH guidelines signed informed consent.

A fixed-dose combination of tenofovir, emtricitabine and efavirenz (TEE) was used for first line treatment. Pharmacy pick up dates for collection of TEE were initially scheduled monthly, then every two to three months once clients were assessed as stable on ART by a clinician. CD4 count was routinely measured at four months and 12 months of ART and viral load at 4 months, 12 months and then annually [15]. All routine laboratory services were provided by the National Health Laboratory Service (NHLS). All ART clients starting ART were registered on the ART routine monitoring system, Tier.net [16]. For sustainability, standard adherence and retention interventions provided by DOH facilities were continued throughout HPTN 071 (PopART), complimented by the work of the CHiPs teams. As per ART guidelines and HPTN 071 (PopART) standard operating procedures, all ART clients should have been supported by clinic-based adherence counsellors, community-based adherence workers (CCWs) and CHiPs teams. The work of the clinic adherence counsellors, CCWs and CHiPs was integrated through joint attendance of clinic operational meetings [14, 15]. Stable ART clients were routinely referred to adherence clubs, either at the facility or in the community, according to DOH adherence club guidelines [17].

### Cohort overview and definitions

This study included data on all clients 18 years and older recorded in Tier.net as initiating ART at the three study clinics, between 1 January 2014 and 30 November 2015 [14, 16]. Follow up continued until 30 May 2016 the time of administrative data censor. Clients with previous ART exposure but no longer on ART, restarting ART at the study clinics were eligible for inclusion in the study sample. Clients transferred into the study clinics from another ART clinic, already on ART, during this period were excluded from the study sample. Baseline CD4 was defined as the most recent CD4 count within the six months prior to starting ART. Baseline TB treatment was defined as having started TB treatment within the 6 months prior to starting ART. Clients were routinely dispensed between 1 and 3 months ART medication at their last recorded clinic visit. The date of their next scheduled visit was calculated based on the number of days medication dispensed. The primary outcome, attrition, was defined as ‘being three months or more late for this calculated next scheduled visit. All clients were followed up for a minimum of six months. Death was not analysed separately due to significant under-recording in Tier.net. Clients electively transferred to another facility were, in line with Tier.net definitions, defined as transfer outs (TFOs).

### Data management

All data were initially extracted from Tier.net except the data on baseline TB treatment which were extracted from the electronic TB register (ETR.net). If the baseline CD4 count results were missing from Tier.net, these were extracted from the National Health Laboratory Services (NHLS) databases. Data from Tier.net were linked to ETR.net data through a matching algorithm utilising name, surname and date of birth in Microsoft SQL Server^TM^. CD4 count data, extracted from the NHLS database, were linked to data in Tier.net using the WC DOH ‘Clinicom number’ as unique clinic identifier in Stata13^TM^. Pharmacy pick up data recorded in Tier.net was used to calculate the date for next scheduled clinic appointment. Data cleaning and validation included cross-referencing data fields within Tier.net and across Tier.net, NHLS and ETR.net databases. Data elements in Tier.net that were adjudged to have with high rates of missing or incorrect data, e.g. baseline WHO stage and data on adherence club attendance were excluded from analysis.

### Analysis

Baseline characteristics were described for continuous and categorical variables and distribution across CD4 strata was assessed using Chi Squared tests and Kruskal-Wallis tests. Incidence rates were estimated and time-to-event analyses were conducted using Kaplan Meier survival and smoothed hazard estimates. Clients were censored on either the date of attrition, TFO, or on 30^th^ May 2016 (end of the study); whichever was the earliest. Unadjusted and adjusted comparisons of the hazard of attrition at different baseline CD4 count strata were carried out using Cox regression. Potential confounding baseline characteristics for inclusion in the adjusted analysis were selected a priori based on clinical relevance; these included: age, sex, pregnancy status, TB treatment, clinic, previous ART exposure of more than 3 months and year of ART start. Baseline CD4 count strata were chosen to align with previous ART guideline cut offs. [18] Proportional hazards assumptions were checked with scaled Schoenfeld residuals. Likelihood ratio tests were used to estimate P values in regression models where categorical variables had more than two strata. For each variable included in the model, the category chosen as baseline for comparison (HR = 1) was based on sample size and clinical significance. Multivariate logistic regression, including the same baseline characteristics, with the exception of baseline CD4 count was used to compare baseline characteristics of clients excluded from analysis due to missing baseline CD4 count and those included in the study sample. All analyses were performed using Stata version 13 (StataCorp LP, College Station, TX, USA).

### Ethics statement

The HPTN 071 (PopART) trial was approved by the Stellenbosch University Health Research Ethics Committee (SU HREC) (Ref. No. N12/11/074) and the London School of Hygiene and Tropical Medicine Research Ethics Committee (Reference number 6362). All clients initiating ART outside local DOH guidelines for HPTN 071 (PopART) gave written informed consent. Further permission for this study and the use of individual level data from the WC DOH sources (Tier.net, ETR.net and NHLS) with a waiver for informed consent has also been received from SU HREC (reference number N12/11/074A), the Western Cape Government (Reference no. WC_2015RP51_715) and City of Cape Town (Reference no. 10529).

## Results

A total of 2593 clients who started ART at the study clinics between 1 January 2014 and end November 2015 were screened for inclusion in the study, of whom 170 (6.6%) were excluded due to missing baseline CD4 counts. This left a sample of 2423 clients included in the analysis. The distribution of clients by baseline CD4 count strata was 631 (26.0%) at CD4 0–200 cells/μL, 708 (29.2%) at CD4 201–350 cells/μL, 582 (24.0%) at CD4 351–500 cells/μL and 502 (20.7%) at CD4 >500 cells/μL. Table 1. Median baseline CD4 count was 328 cells/μL (IQR 195–468 cells/μL). Most clients were women, 1643 (67.8%), and median age was 31 (IQR 26–38) years. One hundred and forty two (8.6% of women) clients were confirmed pregnant at ART initiation. A total of 285 (11.8%) clients were on TB treatment at baseline, this proportion ranged from 25.7% among those with CD4 counts ≤200 cells/μL down to 5.2% among those with counts >500 cells/μL. More clients were treated at metro clinics; 1022 (42.2%) and 947 (39.1%) at Metro 1 and 2 respectively with 454 (18.7%) treated at the rural clinic. A small number of clients, 49 (2.0%) had previous ART exposure of more than three months. The majority of clients started ART in 2015; 1733 (71.5%) compared to 690 (28.5%) in 2014.

**Table 1:** Baseline characteristics.

Baseline factor		Units	0–200 cells/μl*	201–350 cells/μl	351–500 cells/μl	>500 cells/μl	Total	P value**
**All**		N (%)	631 (26.0)	708 (29.2)	582 (24.0)	502 (20.7)	2423	
**Gender**	**Female**	N (%)	355 (56.3)	463(65.4)	421 (72.3)	404 (80.5)	1643 (67.8)	P<0.0001
	**Male**	N (%)	276 (43.7)	245 (34.6)	161 (27.7)	98 (19.5)	780 (32.1)	
**Age**		Median (IQR)	33(29.0–40.0)	31(25.0–37.0)	31(26.0–37.0)	30(25.0–37.0)	31(26.0–38.0)	P = 0.0001
	**18–25 years**	N%	79 (12.5)	179 (25.3)	142 (24.4)	134 (26.7)	534 (22.0)	P<0.0001
	**26–35 years**	N	312 (49.6)	311 (43.9)	272 (46.7)	227 (45.2)	1122 (46.3)	
	**36–45 years**	N	167 (26.5)	138 (19.5)	109 (18.7)	87 (17.3)	501 (20.7)	
	**46–55 years**	N	57 (9.0)	63(8.9)	46(7.9)	44(8.8)	210(8.7)	
	**>55 years**	N	17 (2.7)	17(2.4)	13(2.2)	10(2.0)	57(2.3)	
**Pregnant at ART start**	**Yes**	N	14 (3.9)	39(8.4)	41 (9.7)	48 (11.9)	142 (8.6%)	P<0.0001
**Baseline TB treatment**	**Yes**	N	162 (25.7)	56 (7.9)	41 (6.7)	26 (5.2)	285 (11.8)	P<0.0001
**Clinic**	**Rural 1**	N	88 (13.9)	113(15.9)	126(21.7)	127(25.3)	454 (18.7)	P<0.0001
	**Metro 1**	N	299 (47.4)	301 (42.5)	231 (39.7)	191 (38.1)	1022 (42.2)	
	**Metro 2**	N	244 (38.7)	294 (41.5)	225 (38.7)	184 (36.7)	947 (39.1)	
**Previous ART of > 3mths**	**Yes**	N	27 (4.3)	10 (1.4)	7 (1.2)	5 (1.0)	49 (2.0)	P<0.0001
**ART start year**	**2014**	N	161 (25.5)	208 (29.4)	160 (27.5)	161 (32.1)	690 (28.5)	P = 0.091
	**2015**	N	470 (74.2)	500 (70.6)	422 (72.5)	341 (67.9)	1733 (71.5)	

*Baseline CD4 count categories were chosen to align with ART previous ART guideline cut-offs.

**Chi squared tests were used to evaluate differences across baseline CD4 strata for all categorical variables and the *Kruskal–Wallis test for* numerical variables (age).

The denominator for all %’s is the total sample for that CD4 count category with the exception of pregnancy where the denominator is limited to females in that CD4 count category.

**Fig 1:**
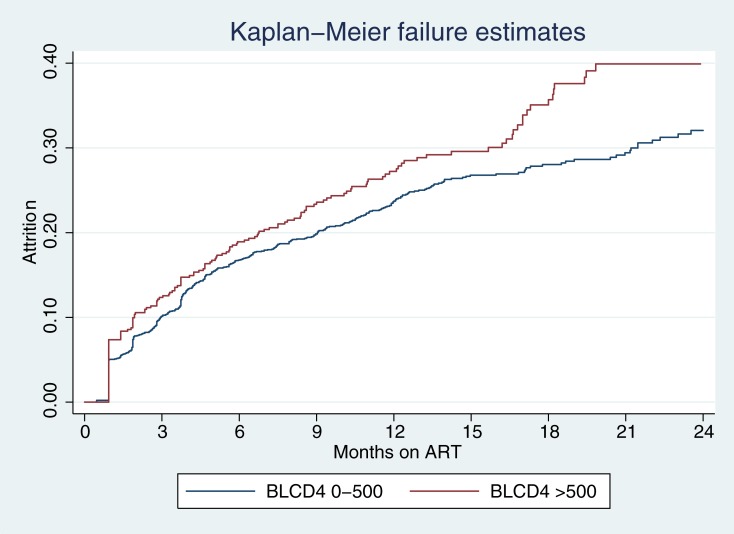
Kaplan Meir failure estimates for attrition stratified by baseline CD4 cell count. BLCD4: Baseline CD4 cell count in cells/μL. Log-rank test for equality of survivor functions: P = 0.02.

Overall, 631 (26.0%) clients experienced attrition during 2389 person years (PY) of follow up (Incidence Rate (IR): 26.4/100 PY) and 140 (5.8%) were TFO. Amongst individuals experiencing attrition, 11 (1.7%) were documented in Tier.net as having died. Median baseline CD4 count amongst those individuals who died was 34 cells/μL (IQR: 63–155). Median follow up time was 11.2 (IQR 7.2–16.1) months. Cumulative numbers of clients experiencing attrition was 418 (17.3%), 561 (23.2%), 613 (25.3%), 631 (26.0%) at 6, 12, 18 and 24 months on ART respectively). Kaplan Meier estimates showed higher attrition amongst clients with baseline CD4 counts > 500cells/μL compared to ≤ 500cells/μL (P<0.02). Fig 1. Attrition peaked at three months across all CD4 count strata and was higher during the first 6 months of ART compared to longer treatment durations; IR: 38.6/100 PY (95% CI 35.1–42.4) from 0 to 6 months ART, 19.2/100 PY (95% CI 16.3–22.6) from 7 to 12 months ART, 14.3 /100 PY (95% CI 10.9–18.7) from 13 to 18 months ART and 11.8 /100 PY (95% CI 7.4–18.7) from 19 to 24 months ART. Table 2.

**Table 2:** Summary of incidence rates of attrition by baseline CD4 count strata and time on ART.

Time on ART	0 to 6 months			7 to 12 months			13 to 18 months			19 to 24 months		
Baseline CD4 strata	PY*	No. LTFU	LTFU/100 PY**	PY	No. LTFU	LTFU/100 PY*	PY	No. LTFU	LTFU/100 PY*	PY	No. LTFU	LTFU/100 PY*
**0–200 cells/μl**	283	108	38.2 (95%CI:31.1–46.1)	194	33	17,1	86	13	15,1	36	5	13,8
						(95%CI:12.1–23.9)			(95%CI:8.8–26.0)			(95%CI:5.7–33.1)
**201–350 cells/μl**	323	119	36,9	223	52	23,3	109	11	10,1	50	8	16,0
			(95%CI:30.1–44.0)			(95%CI:17.8–30.6)			(95%CI:5.6–18.3)			(95%CI:8.0–32.2)
**351–500 cells/μl**	263	96	36,50	184	22	11,9	87	9	10,3	38	2	5,30
			(95%CI: 29.9–44.6)			(95%CI:7.9–18.2)			(95%CI:5.4–19.8)			(95%CI:1.3–21.0)
**>500 cells/μl**	223	95	42,7	156	36	23,1	80	19	23,8	28	3	10,6
			(95%CI:34.9–52.2)			(95%CI:16.7–32.0)			(95%CI:15.2–37.3)			(95%CI:3.4–33.0)
**Total**	1092	418	38,30	756	143	18,9	362	52	14,4	152	18	11,8
			(95%CI:34.8–42.1)			(95%CI:16.1–22.3)			(95%CI:10.9–18.9)			(95%CI:7.5–18.8)

*PY: Person Years

**Incidence rates were calculated using the stptime command in Stata 13^TM^

Adjusted Cox regression analysis using the full model showed higher attrition amongst clients with baseline CD4 counts > 500 cells/μL (aHR 1.26, 95%CI 1.05 to 1.52; (P = 0.014) compared to 0-500cells/μL. Table 3. There was higher attrition amongst clients aged 18–25 years when compared to those aged 26–35 years (aHR 1.30, 95% CI 1.07–1.58). The hazard of LTFU was lower amongst clients starting ART in 2014 compared to 2015 (aHR 0.81, 95% CI 0.66–1.00; P = 0.051). There were no significant differences in attrition between men and women or between study clinics in adjusted analysis.

**Table 3:** Cox regression modelling of baseline characteristics and attrition comparing baseline CD4 categories > 500 cells/μL and 0–500 cells/μL.

		Crude hazard ratio (95% CI)	P	Adjusted hazard ratio (95% CI)	P
**Baseline CD4**	> 500	**1,24(1,03–1,48)**	**0,022**	**1,26(1,05–1,52)**	**0,014**
**(cells/μL)**	0–500	**1**	** **	**1**	** **
**gender**	Male	**1,06(0,9–1,25)**	**0,483**	**1,23(1,03–1,47)**	**0,025**
** **	Femaile	**1**	** **	**1**	** **
**Agecategory**	18–25	**1,29(1,07–1,56)**	**<0.001**	**1,3(1,07–1,58)**	**0,002**
** **	26–35	**1**	** **	**1**	** **
** **	36–45	**0,91(0,73–1,12)**	** **	**0,9(0,72–1,11)**	** **
** **	46–55	**0,7(0,5–0,97)**	** **	**0,69(0,49–0,96)**	** **
** **	>55	**0,82(0,46–1,46)**	** **	**0,8(0,45–1,44)**	** **
**Pregnant at baseline**	Yes	**1,36(1,01–1,82)**	**0,045**	**1,27(0,93–1,72)**	**0,134**
**clinic**	Metro 1	**1**	**0,279**	**1**	**0,137**
** **	Metro 2	**1,05(0,88–1,26)**	** **	**1,16(0,95–1,43)**	** **
** **	Rural 1	**0,89(0,71–1,11)**	** **	**0,96(0,75–1,23)**	** **
**Baseline TB**	Yes	**0,91(0,51–1,61)**	**0,744**	**0,94(0,53–1,68)**	**0,841**
**Previous ART of > 3 mths**	Yes	**0,88(0,5–1,56)**	**0,667**	**0,91(0,51–1,61)**	**0,739**
**Year ART start**	2014	**0,86(0,72–1,02)**	**0,088**	**0,81(0,66–1)**	**0,051**
** **	2015	**1**	** **	**1**	** **

Proportional hazards assumptions were checked with scaled Schoenfeld residuals. Likelihood ratios were used to estimate P values in regression models where categorical variables had more than two strata. Model fits were assessed as good based on the likelihood ratio test statistic. Selection of baseline variable category for comparison (HR = 1) was based on sample size and clinical significance.

Additional multivariate analysis that further stratified baseline CD4 count showed higher attrition in individuals with baseline CD4>500cells/μL when compared to those with baseline CD4 counts 350–500 cells/μL (aHR 1.42, 95%CI 1.12–1.79). S1 Table. There was no significant difference in attrition when comparing clients with baseline CD4 counts 350–500 cells/μL to those with baseline CD4 counts 201–350 cells/μL or 0–200 cells/μL. The higher attrition in individuals with baseline CD4>500 cells/μL persisted in subset analyses restricted to 2281 individuals who were non-pregnant at the time of ART initiation (aHR 1.29, 95%CI 1.07–1.57) S2 Table and in subset analysis restricted to 1100 individuals with follow up of more than 12 months (aHR 1.29, 95%CI 1.07–1.57) S3 Table.

Multivariate logistic regression of factors associated with missing baseline CD4 counts showed increased rates of missing baseline CD4 counts amongst clients who were pregnant at baseline (aOR 2.40, 95% CI 1.35–4.26) and amongst those starting ART in 2014 (aOR 20.2, 95% CI 8.83–46.54) compared to 2015. Being treated at Metro 2 (aOR 0.29, 95% CI 0.12–0.69) and the rural clinic (aOR 0.01, 95% CI 0.00–0.04) were associated with decreased rates of missing baseline CD4 counts.

## Discussion

In this study we found, in a cohort of clients receiving ART regardless of CD4 count at three DOH clinics, we found high rates of attrition, higher amongst clients who started ART with baseline CD4 counts > 500cells/μL compared to those with baseline CD4 count 0–500 cells/μL. This finding was confirmed in subset analyses restricted to individuals non- pregnant at baseline and when restricted to individuals with follow up time of > 12 months. When dividing baseline CD4 count to four strata, attrition was higher amongst clients with baseline CD4 counts > 500 cells/μL compared to those with baseline CD4 counts of 350–500 cells/μL.

The cumulative proportion of clients experiencing attrition in this paper was comparable to that reported by DOH for the corresponding health districts during the same time period [19]; as well as with data from a large systematic review of programmatic data from Africa and Asia (26% at 24 months ART) [20].

This finding of higher attrition amongst individuals with higher baseline CD4 counts, who may be more likely to be clinically well when starting ART, supports findings from previous studies. [7–9]. The underlying reasons for the higher attrition in this group in this study is not clear and may have been driven by psychosocial and health systems factors not measured in the study. The relative novelty of routine provision of ART at baseline CD4 counts >500cells/ μL may also have contributed toward increased attrition in this group. CD4 count is often used in community narratives around ART and it is likely that community perception of the benefits of starting ART at baseline CD4 counts > 500 cells/μL will further develop over time as ART regardless CD4 count becomes standard care. [21].

The extent to which mortality contributed to attrition was not accurately documented in Tier.net. Although mortality as a cause of attrition is decreasing in Africa [22], it remains a significant contributor; particularly during the initial six months on ART [23, 24]. Published studies, with lower median baseline CD4 counts, where clients experiencing attrition were actively followed up with extraction of data from additional sources such as death registries, showed that approximately 40% of clients documented as lost to follow up form ART programmes have in fact died [22]. The START and Temprano RCTs showed significantly lower mortality amongst ART clients with baseline CD4 counts >500 cells/μL [10, 11], and it is therefore plausible that higher attrition amongst those with baseline CD4 counts > 500 cells/μL in this study was driven more by individual choice than by mortality.

The peak in attrition during the first six months on ART across all CD4 strata emphasises the need for additional retention strategies at clinics during early ART. [8, 9, 25–27]The higher attrition amongst younger clients (18 to 25 years) is in keeping with previously published data and highlights the need to continue to strengthen interventions and support focused on this age group [28, 29]. Attrition was also higher amongst clients starting ART in 2015 compared to 2014. This may be associated with overburdening of study clinic resources as a result of increased numbers of clients on ART over time [30, 31]. The WC DOH has a well-established adherence club programme [17] and further development this model of care may be effective in decongesting clinics and improving outcomes [32, 33].

Despite a well-established ART service in the Western Cape since 2004 and provision of ART regardless of CD4 count for HPTN 071 (PopART) since January 2014 a high proportion of clients (26.0%) in this study initiated ART at baseline CD4 counts < 200 cells/μL. This persistence of low baseline CD4, even in the context of ART regardless of CD4 count, is a serious concern. Interventions aimed at promoting earlier ART uptake should therefore continue to be a priority and further strengthened. A recent systematic review of community and clinic based interventions aimed at increasing uptake of ART in sub-Saharan Africa found home based HIV testing and improved efficiencies and structure at clinics to be effective in improving ART uptake [20]. The evidence was, however, reported to be of low quality and the authors identified an urgent need for well-structured studies on the topic. The review also raised concerns about a lack of focus on retention on ART in the literature [20].

### Strengths and weaknesses

The study has a number of key strengths. Data used in the analysis was part of a high quality routine dataset, strengthened by prospective data quality improvement for HPTN 071 (PopART) and clients were provided ART regardless of CD4 count ahead of recent changes to WHO and local guidelines. There were high rates of completeness in key data fields with only a small proportion (6.6%) of eligible clients excluded from the analysis due to missing baseline CD4 count results. There was also high similarity in baseline characteristics associated with attrition between clients excluded due to missing baseline CD4 counts and those included in the study. The three study clinics were typical of metro and rural clinics in the Western Cape and clinic activities were closely aligned to standard care during the study period, supporting generalisability of study findings. Another major strength was the use of an objective measure for determination of attrition (pharmacy pick up date).

There are, however, a number of limitations to consider. Factors not measured by the available date set including psychosocial and health systems factors may have confounded the association between baseline CD4 count and attrition. In this regard; although activities at clinics included in this study were closely aligned for PopART and choice of clinic was not associated with attrition on multivariate analysis it is possible that clinic-related factors not measured in this paper may have confounded the primary analysis. Data and resources required for active follow up of individuals experiencing attrition to determine whether they had died or transferred to another facility without informing their current facility (silent transfers) were not available for this study. Published data show high rates of silent transfer to other ART clinics amongst individuals documented lost to follow up at PHC clinics in the Western Cape. [34] The extent to which silent transfers, contributed to attrition in this study, as in many studies in in high burden regions, is unknown. These silent transfers are likely to have led to overestimation of attrition. Silent transfer may also have been higher amongst clients starting ART at baseline CD4 counts > 500cells/μL, as ART regardless of CD4 counts was not available at neighboring clinics during the study period and clients may have come to the study clinic to initiate treatment and then returned to their ‘usual’ clinic.

Throughout HPTN 071 (PopART) additional support was provided through staff and health systems support at clinics and in the community by CHiPs workers. This support was likely to have reduced overall attrition. Although all ART clients were meant to have received community-based support from both DOH CCWs and/or CHiPs teams, it was not known what proportion of clients received community-based support or whether the community based support differentially impacted clients with baseline CD4 counts > 500cells/μL.

There are further limitations affecting the generalisability of these results. Clients starting ART outside of pertinent DOH guidelines received additional counselling during the signing of research informed consent and which may have in turn reduced their risk of attrition amongst individuals with baseline CD4 >500cells/μL.

## Conclusions

We documented higher attrition amongst clients initiating ART at baseline CD4 counts > 500cells/μL, highlighting an urgent need for retention with a focus on clients initiating ART at higher baseline CD4 counts. At the same time, strategies to improve earlier uptake of ART before their CD4 counts fall below 200cell/μL need to be strengthened. Monitoring systems that more accurately measure the contribution of death and silent transfers to attrition from ART programmes will assist in a better understanding of retention in ART programmes and ART coverage in high burden areas.

## Supporting information

S1 Table Cox regression modelling of baseline characteristics and attrition comparing all baseline CD4 categories. (DOCX)

S2 Table Cox regression modelling of baseline characteristics and attrition comparing baseline CD4 categories > 500 cells/μL and 0–500 cells/μL restricted to individuals non-pregnant at baseline. (DOCX)

S3 Table Cox regression modelling of baseline characteristics and attrition comparing baseline CD4 categories > 500 cells/μL and 0–500 cells/μL restricted to individuals with follow up of >12months. (DOCX)
