# Supplementary material for: Attrition when providing antiretroviral treatment at CD4 counts >500cells/μL at three government clinics included in the HPTN 071 (PopART) trial in South Africa
Source: PLoS One. 2018 Apr 19;13(4):e0195127. doi: 10.1371/journal.pone.0195127 (PMC5909512; doi:10.1371/journal.pone.0195127)
Supplement: S1 Table — (DOCX) [file pone.0195127.s001.docx]

### S1 Table1: Cox regression modelling of baseline characteristics and attrition comparing all baseline CD4 categories.

|  |  | Crude hazard ratio (95% CI) | P | Adjusted hazard ratio (95% CI) | P |
| --- | --- | --- | --- | --- | --- |
| **Baseline CD4**    **(cells/µL)** | **> 500** | 1.39(1.1-1.76) | 0.049 | 1.42(1.13-1.8) | 0.033 |
|  | **351-500** | 1 |  | 1 |  |
|  | **201-350** | 1.2(0.96-1.5) |  | 1.19(0.95-1.48) |  |
|  | **0-200** | 1.16(0.92-1.47) |  | 1.2(0.94-1.52) |  |
| **Gender** | **Male** | 1.06(0.9-1.25) | 0.483 | 1.22(1.02-1.45) | 0.031 |
|  | **Female** | 1 |  | 1 |  |
| **Age**  **category** | **18-25** | 1.29(1.07-1.56) | <0.001 | 1.31(1.08-1.59) | <0.001 |
|  | **26-35** | 1 |  | 1 |  |
|  | **36-45** | 0.91(0.73-1.12) |  | 0.89(0.72-1.11) |  |
|  | **46-55** | 0.7(0.5-0.97) |  | 0.69(0.49-0.96) |  |
|  | **>55** | 0.82(0.46-1.46) |  | 0.81(0.45-1.44) |  |
| **Pregnant at baseline** | **Yes** | 1.36(1.01-1.82) | 0.045 | 1.27(0.93-1.73) | 0.125 |
| **Clinic** | **Metro 1** | 1 |  | 1 | 0.149 |
|  | **Metro 2** | 1.05(0.88-1.26) |  | 1.17(0.95-1.43) |  |
|  | **Rural 1** | 0.89(0.71-1.11) |  | 0.97(0.76-1.25) |  |
| **Baseline TB** | **Yes** | 0.91(0.51-1.61) | 0.744 | 0.92(0.51-1.64) | 0.77 |
| **Previous ART of > 3 months** | **Yes** | 0.88(0.5-1.56) | 0.667 | 0.89(0.5-1.58) | 0.687 |
| **Year ART start** | **2014** | 0.86(0.72-1.02) | 0.088 | 0.81(0.66-1) | 0.046 |
|  | **2015** | 1 |  | 1 |  |

Likelihood ratios were used to estimate P values in regression models where categorical variables had more than two strata. Model fits were assessed as good based on the likelihood ratio test statistic. Selection of baseline variable category for comparison (HR=1) was based on sample size and clinical significance.
