# Supplementary material for: Attrition when providing antiretroviral treatment at CD4 counts >500cells/μL at three government clinics included in the HPTN 071 (PopART) trial in South Africa
Source: PLoS One. 2018 Apr 19;13(4):e0195127. doi: 10.1371/journal.pone.0195127 (PMC5909512; doi:10.1371/journal.pone.0195127)
Supplement: S2 Table — (DOCX) [file pone.0195127.s002.docx]

### S2 Table: Cox regression modelling of baseline characteristics and attrition comparing baseline CD4 categories > 500 cells/µL and 0-500 cells/µL restricted to individuals non-pregnant at baseline.

|  |  | **Crude hazard ratio**  **(95% CI)** | **P** | **Adjusted hazard ratio**  **(95% CI)** | **P** |
| --- | --- | --- | --- | --- | --- |
| **Baseline CD4**  **(cells/µL)** | **> 500** | 1.24(1.02-1.5) | 0.029 | 1.29(1.07-1.57) | 0.009 |
|  | **0-500** | 1 |  | 1 |  |
| **Gender** | **Male** | 1.1(0.93-1.3) | 0.287 | 1.24(1.04-1.48) | 0.017 |
|  | **Female** | 1 |  | 1 |  |
| **Age**  **category** | **18-25** | 1.3(1.06-1.59) | 0.003 | 1.33(1.09-1.64) | 0.001 |
|  | **26-35** | 1 |  | 1 |  |
|  | **36-45** | 0.92(0.74-1.15) |  | 0.91(0.73-1.13) |  |
|  | **46-55** | 0.69(0.5-0.97) |  | 0.68(0.49-0.95) |  |
|  | **>55** | 0.83(0.47-1.49) |  | 0.81(0.45-1.44) |  |
| **Clinic** | **Metro 1** | 1 | 0.225 | 1 | 0.076 |
|  | **Metro 2** | 1.05(0.88-1.26) |  | 1.23(0.98-1.55) |  |
|  | **Rural 1** | 0.86(0.68-1.1) |  | 1.01(0.77-1.31) |  |
| **Baseline TB** | **Yes** | 0.95(0.54-1.68) | 0.86 | 0.97(0.54-1.72) | 0.913 |
| **Previous ART of > 3 months** | **Yes** | 0.97(0.55-1.72) | 0.921 | 1(0.56-1.77) | 0.997 |
| **Year ART start** | **2014** | 0.81(0.67-0.97) | 0.024 | 0.75(0.6-0.94) | 0.011 |
|  | **2015** | 1 |  | 1 |  |

Analysis restricted to 2281 individuals non-pregnant at baseline. Likelihood ratios were used to estimate P values in regression models where categorical variables had more than two strata. Model fits were assessed as good based on the likelihood ratio test statistic. Selection of baseline variable category for comparison (HR=1) was based on sample size and clinical significance.
